# Aged rats are hypo-responsive to acute restraint: implications for psychosocial stress in aging

**DOI:** 10.3389/fnagi.2014.00013

**Published:** 2014-02-12

**Authors:** Heather M. Buechel, Jelena Popovic, Kendra Staggs, Katie L. Anderson, Olivier Thibault, Eric M. Blalock

**Affiliations:** ^1^Blalock Laboratory, Department of Molecular and Biomedical Pharmacology, College of Medicine, University of KentuckyLexington, KY, USA; ^2^Thibault Laboratory, Department of Molecular and Biomedical Pharmacology, College of Medicine, University of KentuckyLexington, KY, USA

**Keywords:** psychosocial stress, aging, cognition, hippocampus, bioinformatics, sleep stages

## Abstract

Cognitive processes associated with prefrontal cortex and hippocampus decline with age and are vulnerable to disruption by stress. The stress/stress hormone/allostatic load hypotheses of brain aging posit that brain aging, at least in part, is the manifestation of life-long stress exposure. In addition, as humans age, there is a profound increase in the incidence of new onset stressors, many of which are psychosocial (e.g., loss of job, death of spouse, social isolation), and aged humans are well-understood to be more vulnerable to the negative consequences of such new-onset chronic psychosocial stress events. However, the mechanistic underpinnings of this age-related shift in chronic psychosocial stress response, or the initial acute phase of that chronic response, have been less well-studied. Here, we separated young (3 month) and aged (21 month) male F344 rats into control and acute restraint (an animal model of psychosocial stress) groups (*n* = 9–12/group). We then assessed hippocampus-associated behavioral, electrophysiological, and transcriptional outcomes, as well as blood glucocorticoid and sleep architecture changes. Aged rats showed characteristic water maze, deep sleep, transcriptome, and synaptic sensitivity changes compared to young. Young and aged rats showed similar levels of distress during the 3 h restraint, as well as highly significant increases in blood glucocorticoid levels 21 h after restraint. However, young, but not aged, animals responded to stress exposure with water maze deficits, loss of deep sleep and hyperthermia. These results demonstrate that aged subjects are hypo-responsive to new-onset acute psychosocial stress, which may have negative consequences for long-term stress adaptation and suggest that age itself may act as a stressor occluding the influence of new onset stressors.

## Introduction

Normal aging is a complex process resulting in functional decline and increased susceptibility to a variety of insults across multiple organ systems. The US Census Bureau predicts that the aging population will triple by 2050, dramatically increasing aging and age-related disease burden on health care infrastructure in the United States (Hebert et al., 2013). Brain tissue represents a critical point of failure, with reduced quality-of-life and autonomy, and increased susceptibility to neurodegenerative disease (Tornatore et al., 2003). Intense research over the past 40 years has forwarded several putative mechanisms of aging-related neurologic dysfunction including free radical damage (Finkel and Holbrook, 2000; Cutler et al., 2004; Poon et al., 2004), protein misfolding (Wickner et al., 1999; Soto, 2003), calcium ion dyshomeostasis (Landfield, 1987; Khachaturian, 1989; Landfield et al., 1992; Foster et al., 2001; Toescu and Verkhratsky, 2004; Thibault et al., 2007; Toescu and Vreugdenhil, 2010), and stress/stress hormone exposure (allostatic load/glucocorticoid cascade) (Landfield, 1978; Landfield et al., 1978b, 2007; Sapolsky et al., 1986b; Kerr et al., 1989; Lupien et al., 1998; Porter and Landfield, 1998; McEwen, 1999; Barrientos et al., 2012).

The hippocampus plays a critical role in cognitive function (Lupien et al., 1998; Yankner et al., 2008) and provides important feedback regulation over the hypothalamic-pituitary-adrenal (HPA) axis (McEwen et al., 1992; Rostene et al., 1995; Ziegler and Herman, 2002; Joels et al., 2013). HPA axis activity is associated not only with stress, but with sleep and circadian rhythm (Plihal and Born, 1999; Garcia-Borreguero et al., 2000; Van Cauter et al., 2000). Synergistic actions of stress and aging are thought to be especially disruptive in the hippocampus, leading to memory impairments and weakened control over stress hormones (Sapolsky et al., 1984; Meaney et al., 1992; Stranahan et al., 2008). The convergence of age and stress at the hippocampus is highlighted by evidence of increased cognitive dysfunction in aged humans after exposure to new-onset stress, including jet lag, physical wounding, anesthesia, infection or psychosocial stressors (PS- stressors that do not involve nociceptive input) (Wofford et al., 1996; Bekker and Weeks, 2003; Lupien et al., 2005; Vondras et al., 2005; Barrientos et al., 2012).

Extensive work with animal models clearly shows that early life (neonatal, prenatal) stress exposure has life-long deleterious consequences (Meaney et al., 1988; McEwen et al., 1999; Weaver et al., 2006; Loria et al., 2010; Harris and Seckl, 2011), and that glucocorticoids, stress exposure, or manipulations that promote stress signaling in young subjects recapitulates aspects of the aging phenotype (Wei et al., 2007), including impairment on spatial and working memory tasks (Wright et al., 2006; Ferrari and Magri, 2008; Barsegyan et al., 2010; Marin et al., 2011), enhancement or preservation of emotional memory (McGaugh and Roozendaal, 2002), neuronal functional deficit (Kerr et al., 1989, 1991, 1992; Krugers et al., 2012), transcriptional change (Porter et al., 2012; Chen et al., 2013), and disrupted sleep architecture (Pawlyk et al., 2008). Interestingly, prior stress exposure blunts response to a subsequent stressor (adaptation) in young animals (Paskitti et al., 2000; McEwen, 2001), but not in aged animals (Spencer and McEwen, 1997), suggesting aging alters the brain response to PS.

New onset PS is highly prevalent in aged humans and has negative consequences for sleep and cognition (House et al., 1990, 1994). However, basic research on these aging responses has lagged behind (Maines et al., 1998; Lupien et al., 2009; Porter et al., 2012). Here, we designed experiments to establish whether aged subjects responded differently than young to acute PS. Restraint, an animal model of PS (reviewed in Buynitsky and Mostofsky, 2009) was applied to young (3 month) and aged (21 month) male F344 rats. Sleep architecture, water maze performance, blood glucocorticoid level, circadian temperature oscillations, hippocampal electrophysiology and gene expression (NanoString evaluation of a panel of 200 previously defined aging sensitive hippocampal genes) were measured. Aged animals compared to young, as well as young animals responding to stress exposure, showed responses typical to those seen in prior work. However, aged animals showed either a weaker response, or a lack of response compared to their younger counter parts on sleep, maze, body temperature, and blood glucocorticoid measures. This reduced response suggests, among other possibilities, that aging itself may act as a stressor, occluding the acute PS response. Our results raise the intriguing possibility that failure to respond to an acute stress may have long-term negative adaptive consequences for aged subjects.

## Materials and methods

### Subjects

Young adult (3 month), *n* = 21 and aged (21 month) *n* = 19 males Fischer 344 rats obtained from the NIA aging colony were individually housed with enviro-dry paper bedding, a rat tunnel and a Nyla bone. Animals were maintained on a reverse 12:12 light/dark (4:30 AM lights off, 4:30 PM lights on) and were given access to food and water *ad-libitum*. Additional subjects not included in the analysis were excluded based on pathology (1 young, 4 aged), surgical complications (2 young, 5 aged), or failure to reach behavioral criteria (0 young, 2 aged). All experiments were performed in accordance with institutional and national guidelines and regulations, and conform to our approved protocol (University of Kentucky IACUC #2008-0347).

### Surgery

All subjects were implanted according to standard procedures with wireless EEG/EMG emitters (Data Sciences International- TL11M2-F40-EET) as in prior work (Buechel et al., 2011). Prior to surgery, EEG wires were cut to length and a sterile 1/8” stainless steel screw was soldered to the end of each lead. To begin surgery, animals were anesthetized with isoflurane and placed in a stereotaxic frame. A two inch incision was made to expose the skull and spinotrapezius muscles. The emitter was placed under the skin between the left scapulae and the left ileum along the flank. The exposed dorsal region of skull was cleaned with 3% peroxide and the skull surface dried with sterile cotton swabs soaked in 70% ethanol. For EEG electrodes, a 0.7 mm hole was drilled 1 mm from either side of the sagittal suture line and 1–2 mm anterior to the lambda suture line. Screws were inserted into the holes and positioned so that the flat screw tip rested on the dura. Screw heads were covered with dental cement and left to dry. EMG electrodes were inserted through the trapezius muscle with a 21guage needle, perpendicular to the muscle fibers. The free wire end was capped with insulation and both sides of the incision were tied off with surgical thread to prevent fluid infiltration. The incision was then closed with 6–8 mattress stitches.

### Sleep data acquisition and analysis

Animals were housed individually and cages were positioned at least 18” apart to avoid interference during radiotelemetry data acquisition. EEG, EMG, temperature and locomotor activity data were recorded continuously with DSI's Data Art acquisition software and binned in 10 s epochs. For these nocturnal rodents, the first 4 h of their active period (dark) and the first 4 h of their resting period (light) were evaluated for sleep architecture on the day prior to the start of water maze training (baseline), and following the stress/probe trial paradigm. Architecture was scored using Neuroscore's analysis console in 30 s increments while being viewed in 2–5 min windows. EEG waves were stratified into “low amplitude” (≤50% of maximum) and “high amplitude” (>50% of maximum) tiers, and underwent fast Fourier transforms for each of 5 frequency ranges: Δ(0.5–4 Hz), Θ (4–8 Hz), A (8–12 hz), Σ (12–16 Hz), and B (16–24 Hz). EMG waves were stratified into 3 tiers: “basal” ≤33% (seen during REM), “intermediate” (between 33 and 66%), and “high” (>66%). Stages based on EEG/EMG signaling were established as follows: Wake- intermediate or high EMG ± locomotor activity, EEG variable; Light Sleep- low amplitude EEG, intermediate EMG, and no locomotion; REM (paradoxical) Sleep- high frequency EEG, “basal” EMG and no locomotor activity; Deep Sleep- high amplitude EEG activity enriched in delta band frequency, basal to light EMG activity, no locomotor activity. Prior assigned sleep stages informed subsequent assignments. Ambiguous epochs, as well as those containing artifacts, were not scored and accounted for <5% of scored time.

### Water maze testing

The water maze task was performed as in previous studies (Buechel et al., 2011). A 190 cm diameter circular, black painted pool was centered (250 cm/side) in a cubicle of floor to ceiling black curtains, making the environment relatively neutral. High contrast black and white cues (90 × 90 cm- circle, triangle and vertical lines), were placed, one to each of three curtains facing the maze, 60 cm above the maze rim. Maze temperature was maintained at 26 ± 2°C. One quadrant contained a 15 cm diameter escape platform covered with black neoprene for improved traction. Illumination in the room was set at 3.6 to 3.8 lux and a Videomex-V water maze monitoring system (Columbus Instrument, Columbus, OH) was used for analyses. All training and probe sessions took place between 12PM and 4PM (during the rats' active period).

#### Locally cued training (pre-surgery)

The locally cued platform location task included an additional visual cue: a white Styrofoam cup suspended from the ceiling by black thread approximately 12 inches above the submerged platform. In the locally cued task, over 3 days, each animal was given three 60 s trials per day, with 60 s on the platform and a 2 min inter-trial interval. Criterion for performance was established as an ability to swim to the platform in under 30 s for 2/3 trials on the 3rd day. The spatial cues destined to be used in the spatial training task were already mounted during locally cued training.

#### Spatially cued training and probe (post-surgery)

Two weeks after implantation surgery (to allow for recovery), the spatial water maze task was performed as in prior work (Buechel et al., 2011). Spatial cues outside the pool were provided. A 4 day protocol was used (days 1–3: 3 trials per day, submerged platform (~2 cm below water surface); day 4-following restraint- probe trial with platform removed). On training days, each animal began in a different quadrant on each of three trials. They were given 1 min to find the platform, 1 min on the platform and a 2 min inter-trial interval. On the probe day, the platform was removed and each rat was given one 60 s trial. Platform position was constant throughout local, spatial and probe trials, but starting quadrant was changed for each trial. For probe trial, animals always started in quadrant opposite to goal quadrant.

### Restraint (psychosocial) stress

For 3 h immediately prior to the water maze probe trial, half of the subjects were restrained with nylon coated canvas rat Snuggles® (Harvard apparatus). Animals were monitored continuously for vocalization and struggling throughout the 3 h restraint, and tested on the probe trial immediately following restraint. Control animals stayed in their home cages in the housing facility until the probe trial began. After completing the task, animals were returned to their home cages in the housing facility and sleep architecture data was collected.

### Tissue collection and analysis

On the following morning, animals were killed by CO_2_ anesthesia and decapitation. Trunk blood was collected in lithium heparin vacutainers (BD biosciences), and centrifuged at 1200 g for 10 min. Serum was removed for corticosterone measurement using a radio-immuno assay with a lower quantification limit of 20 ng/ml (Antech GLP, Siemens Diagnostic, Los Angeles) and additional blood components measured (Abaxis Comprehensive Diagnostic Panel, VetScan2,University of Kentucky College of Medicine core facility). The hippocampus was dissected out of one hemisphere and prepared for electrophysiological testing. The anterior and posterior tips of those hippocampi were placed in dry ice and transferred to a −80°C freezer for NanoString analysis at the end of the study. The second hemisphere was post-fixed in 4% formalin (24 h), cryoprotected in 15% sucrose solution and stored at −80°C for future use.

### Electrophysiology

Hippocampi were prepared according to standard protocols (Searcy et al., 2012; Pancani et al., 2013). Briefly, hippocampi were removed and cut into transverse 400 μm thick slices on a McIlwain chopper. They were placed in low calcium artificial cerebrospinal fluid (ACSF) chilled to 0°C, recipe as follows (in mM): 128 NaCl, 1.25 KH_2_PO_4_, 10 glucose, 26 NaHCO_3_, 3 KCl, 0.1 CaCl_2_, 8 MgCl_2_. Slices were transferred to an interface recording chamber kept at 32°C with 95% O_2_, 5% CO_2_ and normal-calcium ACSF (as above) with CaCl_2_ increased to 2 mM and MgCl_2_ reduced to 2 mM. Slices were allowed to incubate in the recording chamber for at least 1 h before recording.

To begin testing, a 0.0055 inch, twisted bipolar Teflon coated stainless steel (A&M Systems) stimulating electrode, was placed in the perforant pathway. Glass recoding electrodes, pulled on a Sutter-Brown P-80 puller with tips broken back (2–4 MΩ resistance), were filled with ACSF and placed in the *stratum radiatum* of CA1 to record excitatory postsynaptic field potentials (EPSPs). We performed 2 experiments on each slice. First, input/output curves were constructed by varying input stimulator voltage and measuring change in output EPSP. Then input voltage was set to generate 1/3 of maximum EPSP and paired pulse (PP) facilitation experiments were performed in triplicate with 50, 100, 150, and 200 ms delays and a 30 s inter-trial interval.

### Gene expression analysis

Microarray data from three published studies of hippocampal aging in the F344 rat (Blalock et al., 2003; Rowe et al., 2007; Kadish et al., 2009) (data available through the Gene Expression Omnibus GSE854, GSE5666, GSE9990) was compiled to establish lists of genes significantly upregulated (101 total) or downregulated (70 total) in at least 2/3 of these studies. This panel of “male F344 rat hippocampal aging genes” was submitted to NanoString Technologies (Seattle, WA) who then constructed a custom nCounter multiplex code set (Geiss et al., 2008) for the detection of these mRNA species (and specifically, for the gene structures as assayed by the original Affymetrix microarray probe set designs). Hippocampal mRNA was extracted from fresh frozen hippocampal tissue as in as in prior work (Buechel et al., 2011; Porter et al., 2012). Briefly, the dorsal and ventral tips of hippocampi were placed in Eppendorf tubes, frozen on dry ice, and stored at −80°C. After all tissue was collected, RNA was extracted using the Trizol protocol and RNA quality was checked on Agilent Bioanalyzer (RNA Integrity Number-RIN: 7.27 ± 0.15). 171 pre-selected mRNA species were quantified with nCounter (12 animals- 3 per treatment group- were run on each nCounter plate, except the last, which measured 6 subjects- 4 young controls, 1 aged stress, and 1 young stress). Five subjects' nCounter data was excluded due to poor quality). Data from the remaining 37 subjects (*n* = 10 young control, 8 young stress, 10 aged control, 9 aged stress) were transferred to flat files for further analysis. Data were normalized according to standard nCounter procedures using NanoString provided spike-in controls. Quality control measures were consistent across groups and within normal range (binding density: 0.6 ± 0.04; Fields of View Read: 599.7 ± 0.1; n.s. between treatment groups, two way ANOVA). None of the probes selected failed to detect a signal. Complete list of results is provided (Supplemental Table 1).

### Statistical analysis

Data were analyzed by conventional statistical procedures as noted in Results with α = 0.05. *P*-values are reported in figure captions if they are significant and listed as “n.s.” if non-significant. Asterisks and arrows/lines are used to highlight significant *post hoc* pairwise results. Multiple testing error was estimated where appropriate using the False Discovery Rate procedure (Hochberg and Benjamini, 1990). Software used for statistical analysis included Excel (Microsoft, 2010), SigmaStat (v 3.5, Systat), and The Institute for Genomic Research's Multi-Experiment Viewer (Saeed et al., 2006).

## Results

### Water maze

**Figure 1:**
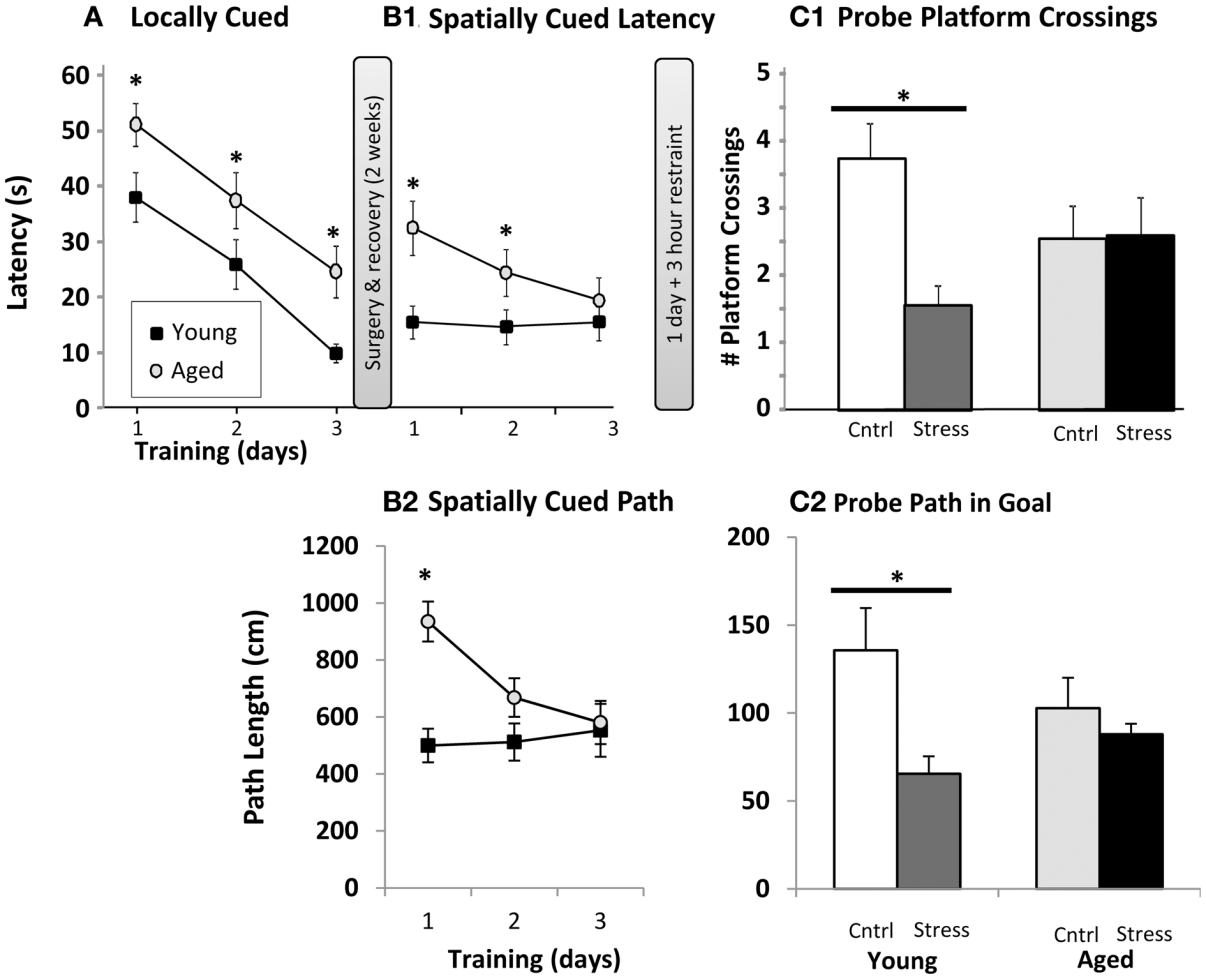
**Water maze. (A)** Locally cued training. Latency to platform is plotted as a function of training day (*p* < 0.001 for main effects of training and age, interaction n.s., two way repeated measures ANOVA; ^*^p ≤ 0.05 for age at each training day *post hoc* pairwise Fisher's LSD). **(B1)** Spatially cued training latency plotted as in **(A)**. Main effects of age (*p* < 0.001) and training (*p* < 0.01), as well as interaction (*p* < 0.01) are significant by two way repeated measures ANOVA (^*^ as in **A**). **(B2).** Spatially cued training path lengths are plotted (age *p* < 0.01, training *p* < 0.05, interaction *p* < 0.01, ^*^ as in **A**). **(C1).** Probe trial platform crossings. The number of times the original platform location was crossed (# platform crossings) is plotted for young and aged animals that were control (Cntrl) or 3 h restraint treated (Stress). Main effects of age and stress were not significant, but interaction was significant (*p* < 0.05) by two way ANOVA. (^*^p ≤ 0.05, Fisher's LSD young control vs. young stress). **(C2)** Probe trial path length (in cm) within the goal quadrant. Main effects of age and stress were not significant, but interaction was significant (*p* < 0.05) by two way ANOVA. (^*^p ≤ 0.05, Fisher's LSD young control vs. young stress).

Animals were initially trained to swim to a platform centered immediately under a visual cue (“locally” cued). The purpose of this task was to identify and remove subjects that could not see or were too distressed to perform the task. Over three successive days, latency to the platform was measured on three trials per day. Results are averaged and plotted (Figure 1A). Both young and aged animals showed shorter latencies over time, but aged animals were significantly slower than young on all training days. Next, subjects were surgically implanted with sleep-monitoring equipment and given 2 weeks for recovery (Methods) and baseline sleep measurement.

After recovery, the local cue was removed from the water maze and animals were trained, 3 trials per day for 3 days, on the spatial Morris water maze. Here, distal cues are used to triangulate on the hidden platform's location. Young animals appeared to be performing at peak levels, measured as either latency (Figure 1B1) or path length (Figure 1B2), on all training days, while aged subjects continued to improve. Because the spatial cues were present during the locally cued task and platform location was constant, an intriguing possibility is that the young animals acquired spatial cues during the locally cued task, but aged animals did not, or at least not to the same degree as the young. Aged animals did, however, show significantly shorter latencies over training days (i.e., continued improvement on the task). Aged subjects were significantly slower on training days 1 and 2, but statistically indistinguishable from young by training day 3 (Figure 1B1). Path length (Figure 1B2) revealed a similar pattern albeit young vs. aged significance was only present on the first training day. This allowed us to evaluate the effect of 3 h restraint from a similar performance baseline in young and aged subjects. It should be noted that aged animals swam more slowly than young (young 33.7 ± 1.1 cm/s; aged 28.5 ± 0.9 cm/s; *p* < 0.01), and this reduced speed could in part explain longer latencies in aged subjects. However, with an average distance from all drop points to platform of 91.44 cm, young and aged subjects' minimum “straight line” latency to platform, given their average swim speeds, would be 2.7 s and 3.2 s respectively. Therefore, swim speed alone accounts for about 0.5 s difference in latencies, a performance gap much smaller than observed on locally cued, and days 1 and 2 of spatially cued, training.

On the following day, half of the animals were restrained for 3 h, and the hidden platform was removed from the maze for probe trial. During a 60 s swim period, we counted the number of times each animal crossed the area originally containing the platform (Figure 1C1), as well as the animals' path length within the goal quadrant (Figure 1C2). As in prior work, aged animals dedicated less effort to investigating the original platform location. However, while 3 h of restraint significantly reduced young animal's platform crossings and goal quadrant path lengths, an effect seen in multiple other restraint studies, aged subjects appeared insensitive to restraint. Similar, albeit borderline significant, patterns (*p* = 0.06–0.1) were seen with swim latency and path length to platform location (data not shown).

### Restraint stress

For the 3 h immediately preceding the spatial water maze probe trial on the 4th day, half of the young and aged animals were restrained. Animals were monitored throughout the restraint period for vocalizations and struggles. Qualitatively, both young and aged animals appeared distressed by the procedure. Quantitatively, we were unable to detect any age-related difference in perceived stress as measured by the number of struggles and vocalizations over 3 h (Young 29.6 ± 7.9, Aged 29.6 ± 7.1; n.s., Student's *t*-test).

### Sleep architecture

**Figure 2:**
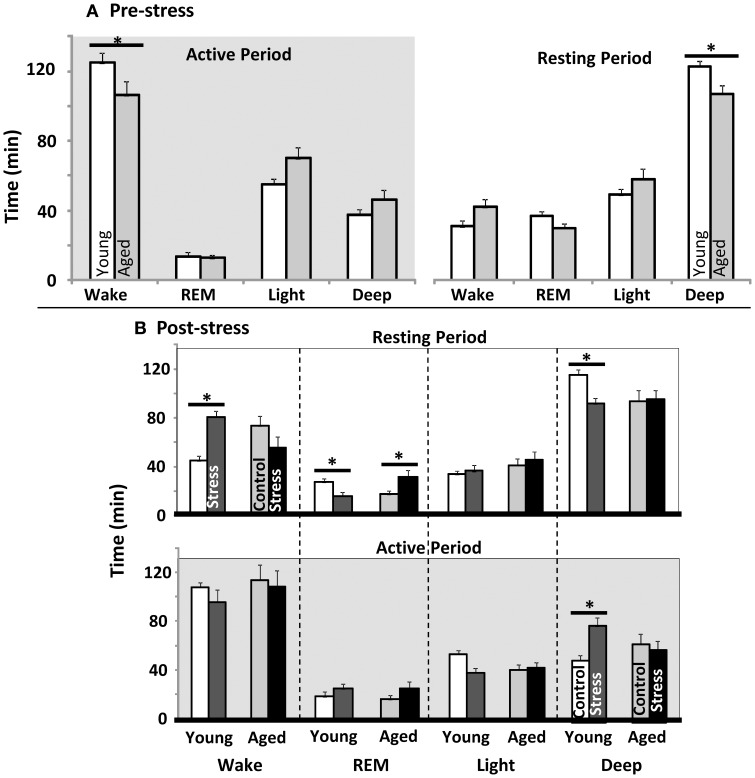
**Sleep architecture pre and post water maze. (A)** Young vs. aged sleep stage duration before stress or water maze exposure. Total minutes in each sleep stage during the first 4 h of the active as well as rest periods are plotted (*p* < 0.001 for stress, aging n.s., and *p* < 0.05 for interaction between stress and aging in both Active and Resting periods; two way repeated measures ANOVA). **(B)** Influence of stress exposure on young and aged sleep architecture: (Top) Resting period immediately following stress and (Bottom) during subsequent active period (12 h later). Each sleep stage in each period was tested separately by two way ANOVA. No main effects for any stage were significant. However, the Interaction term was significant for resting period: wake (*p* < 0.05), REM (*p* < 0.05) and Deep (*p* < 0.01); as well as Active: Deep (*p* < 0.01). (For all panels ^*^p ≤ 0.05, *post hoc* pairwise Fisher's LSD).

EEG and EMG data were scored for duration of Wake, REM sleep, light sleep, and deep sleep. The first 4 h of Resting and Active periods were evaluated before spatial water maze training, and again after restraint stress. As in prior work, control aged subjects showed less active period wake and less resting period deep sleep than young (Figure 2A). Restrained young animals showed significantly more wakefulness, and less REM and deep sleep during the inactive period, while aged animals showed only elevated REM sleep (Figure 2B upper). During the post-stress active period, young rats only showed a significant elevation in deep sleep (Figure 2B lower). Note that previous work has shown, in both humans and rodents, that increased active period deep sleep is related to poor cognition (Reid et al., 2006; Buechel et al., 2011)

### Body temperature

**Figure 3:**
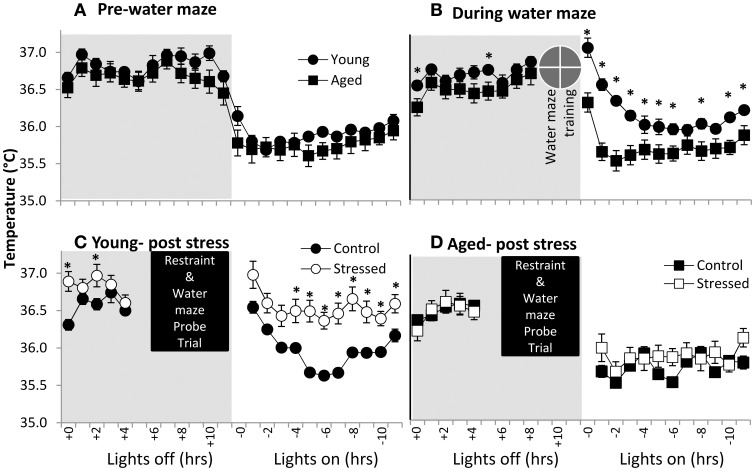
**Body temperature in young and aged. (A)** Pre-water maze baseline temperature measures show no difference between young and aged subjects. (time *p* < 0.001, age n.s., interaction n.s.; two way repeated measures ANOVA). **(B)** During water maze training, young subjects show extended hyperthermia in the resting period after water maze exposure. (time *p* < 0.001, age *p* < 0.005, interaction *p* < 0.001; two way repeated measures ANOVA; ^*^p ≤ 0.05 for young vs. aged at time *x*). **(C)** Young subjects post-stress show elevated temperature. (time *p* < 0.001, stress *p* = 0.01, interaction n.s.; two way repeated measures ANOVA; ^*^p ≤ 0.05 for control vs. stressed at time *x*). **(D)** Aged subjects show no statistically significant post-stress hyperthermia. (time *p* < 0.001, stress n.s., interaction n.s.; two way repeated measures ANOVA).

Body temperature is well understood to be diurnally regulated (Adan et al., 2012; Menaker et al., 2013) as well as responsive to stress (stress-induced hyperthermia- SIH) (for review see Adriaan Bouwknecht et al., 2007). Although not well-defined, prostaglandin signaling, the same system responsible for the fever response in illness, may participate in SIH (Oka et al., 2001). In the present work, body temperature data were acquired through the surgically implanted telemetry devices and used to evaluate potential age-related differences in diurnal regulation and SIH. Aged and young subjects are clearly in sync with light cues prior to spatial water maze training (Figure 3A- the average of three 24 h periods of days of baseline recording), suggesting similar diurnal regulation in young and aged subjects.

To investigate SIH, we measured body temperature in response to both spatial water maze training and restraint (Figures 3B–D). Clearly, young animals show elevated resting period body temperatures in response to spatial water maze training (Figure 3C- average of 3 days training), although lights on/lights off responses remain synced. Aged animals' body temperatures during water maze training are similar to their control levels. Finally, young animals show an exacerbated temperature elevation (Figure 3C) following restraint, while aged animals' body temperature appears relatively unresponsive to restraint (Figure 3D).

### Hippocampal electrophysiology

**Figure 4:**
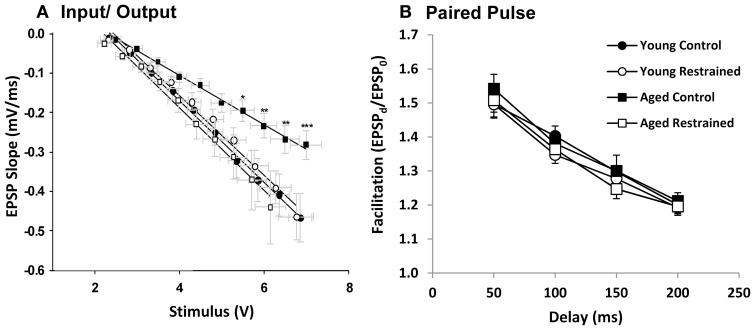
**Electrophysiology. (A)** EPSP slope plotted as a function of stimulus voltage for each age and restraint condition. For statistical testing, stimulus voltage was categorized (i.e., voltage steps 1–10). By two way repeated measures ANOVA, stimulus significantly influenced slope (*p* < 0.001), while there was no significant group effect. However, interaction was significant (*p* < 0.05). *Post hoc* pairwise results (Fisher's LSD; ^*^p ≤ 0.05, ^**^p ≤ 0.01, ^***^p ≤ 0.001) contrast young control and aged control and reveal significant deflection of aged control EPSP slope especially at steps 7–10. **(B)** Paired pulse facilitation plotted as a function of delay for each age and restraint condition. There was a significant effect of delay (*p* < 0.001) but not group or interaction (two way repeated measures ANOVA).

Animals were killed the day following restraint and hippocampal slices were prepared for extracellular synaptic recordings. Input/output (I/O) experiments assessed the relationship between synaptic response (output) and stimulus (input) (Figure 4A). Aged control animals showed a significantly weakened input/output relationship, as has been seen in prior studies (Barnes and McNaughton, 1980; Landfield et al., 1986). This weakening appeared, at least in part, recovered in stressed aged subjects. We also tested for presynaptic calcium perturbations using the PP facilitation (Figure 4B) protocol. As in previous studies (Landfield et al., 1978a; Wu and Saggau, 1994), no age-related changes were detected. Further, no change was seen in either of these measures with stress exposure.

### Blood corticosterone (CORT)

**Figure 5:**
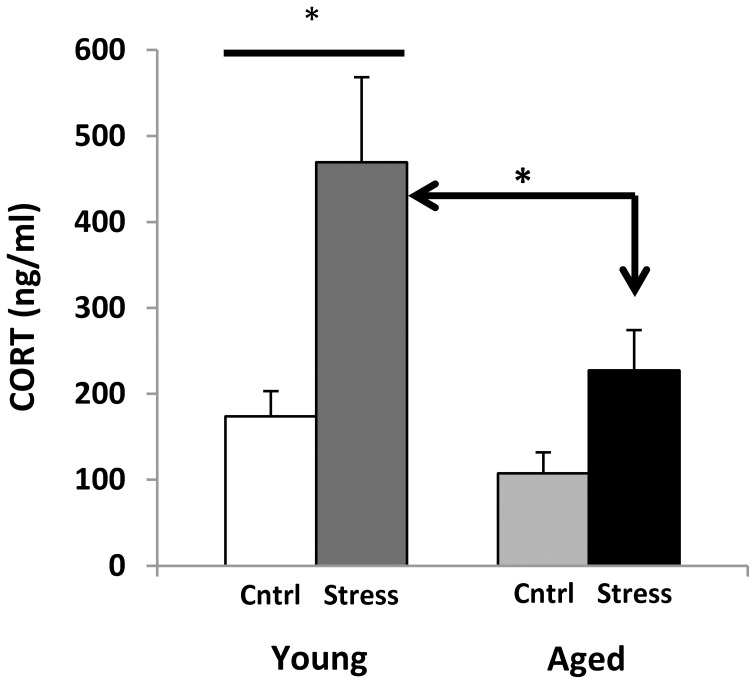
**Corticosterone levels.** CORT measures for young and aged rats are plotted for control and stress exposure. Both stressed groups show an increase in CORT. Two way ANOVA reveals a significant main effect of Age (*p* = 0.01) and Stress (*p* < 0.001), and no significant interaction. *Post hoc* Fisher's LSD pairwise comparisons (^*^p ≤ 0.05) show a significant contrast between control and stress CORT levels in young but not aged animals, as well as significantly elevated CORT levels in young stressed vs. aged stressed animals.

Corticosterone radio-immunoassays were performed on trunk blood that was collected at decapitation (during active period, 21 h following restraint). However, all animals were transported on the procedure day, and were killed by CO2 anesthesia, and both procedures are known to elevate CORT (Vahl et al., 2005). Despite this, young animals that had been restrained showed much higher blood CORT (Figure 5), while aged subjects showed a blunted response. Future studies monitoring CORT levels throughout the stress exposure paradigm may help to discern whether this phenomenon might represent “adrenal exhaustion” in the aged subjects.

### Hippocampal gene expression

**Figure 6:**
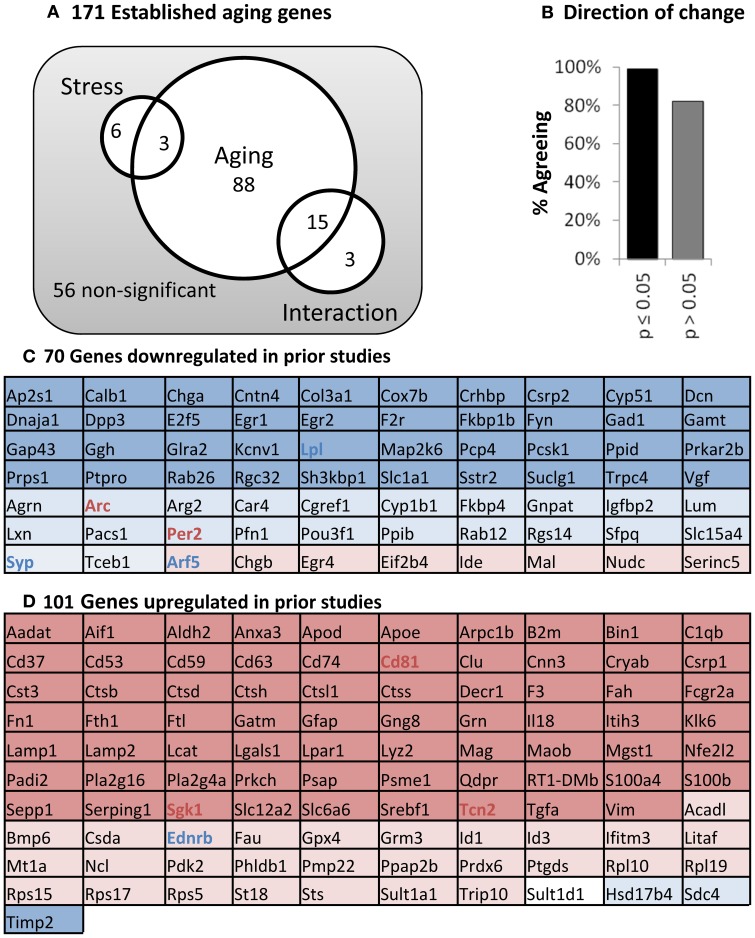
**Panel of aging genes. (A)** Venn diagram. Of 171 established aging genes, 115 are changed significantly in this study (two way ANOVA; significant main effects of aging or stress, or interaction *p* ≤ 0.05). **(B)** Direction of Change. Nearly complete agreement with prior studies regarding direction of change for aging-sensitive genes. **(C)** Genes down-regulated in previous studies. **(D)** Genes up-regulated in previous studies. Color: Blue, down; Red, Up, Shading; Dark, age significant; light, age non-significant; Bold, stress-significant.

Hippocampal mRNA was tested for aging and stress changes against an “aging” panel of 171 genes found to change significantly with age in at least two of three published hippocampal aging microarray studies in male F344 rats (see Methods). As predicted, far more genes are significant than could be reasonably explained by multiple testing error (115 found vs. 9 expected with 171 tests at α = 0.05) and are primarily centered on aging (Figure 6A). Additionally, nearly all significant genes in the present study also agreed in direction of change with prior work on aging (Figure 6B). Even non-significant genes showed >80% agreement in direction of change with prior aging studies (*p* < 0.001, binomial test). Genes altered with age were largely associated with results as reported in prior work, including down-regulated neuronal and synaptic markers, and up-regulated immune and inflammatory signaling. All 171 genes in the aging panel are shown in Figure 6C (for genes previously defined as up-regulated with age) and 6D (for genes previously defined as down-regulated), and color-coded with their results in the present study (complete descriptions are available in Supplemental Table 1).

**Figure 7:**
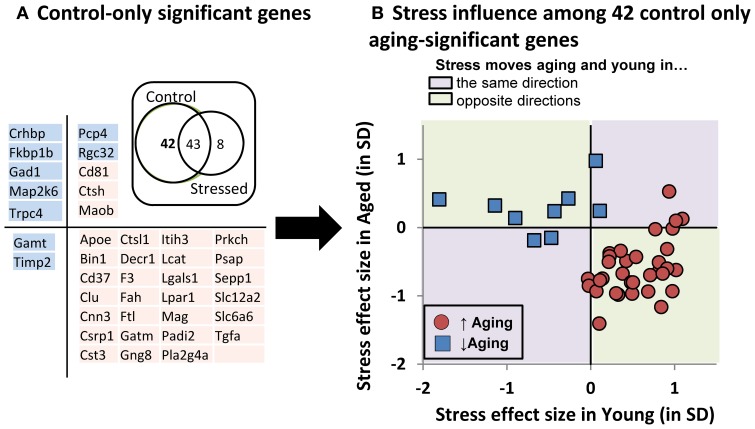
**Stress influence on control-only aging-significant genes. (A)** Table of control-only significant genes separated by directional influence of stress within young and within aged subjects. (**A**-inset): Venn diagram of pairwise contrast results- a majority of aging-significant genes were significant in both control and stressed groups. However relatively few genes were selectively changed with age in the stress condition. Instead, control-only aging significant genes appeared relatively enriched. **(B)** Stress' influence on age-related gene expression. Individual gene expression stress effect sizes [(stress mean–control mean)/pooled SD] within young (x axis) or within aged (y axis) subjects is plotted.

To better depict stress' influence on the aging transcriptional profile, we performed pairwise contrast analyses (Figure 7A- Inset Venn). A large cohort of genes (43) significantly changed with age regardless of stress status, but relatively few genes (8) changed with age in the stress-only condition. In contrast, a large group of genes (42) changed with age in control, but not stressed, subjects (listed in 7A). Taken together, these data suggest that young and aged subjects' transcriptional profiles become more similar to one another under stress conditions.

We plotted the “young control to young stress” effect size on the x axis against the “aged control to aged stress” effect size on the y axis for these 42 “stress-blunted” aging genes (Figure 7B). The majority (33/42 = 79%) showed oppositional regulation in young vs. aged subjects. Exclusively up-regulated genes (red symbols in lower right quadrant of Figure 7B) show increased expression in young that have been exposed to stress (stress shifting gene expression in an aging-like direction) and decreased expression in aged that have been exposed to stress (stress shifting gene expression in a young-like direction), effectively constricting the magnitude of aging's influence on the expression of these genes. A similar, albeit less pronounced, phenomenon is seen for down-regulated genes. Because the magnitude of these effects are not significant for individual genes and seem more meaningful as an aggregate, we reserve speculation on these changes for the Discussion.

## Discussion

Despite clinical data showing both an increased incidence of, and more deleterious response to, chronic psychosocial stress with age in humans, surprisingly little basic research has investigated psychosocial stress' potential negative influences on sleep and cognition in aged subjects. Here, we determined whether age was a factor in acute psychosocial stress response across multiple measures. In our hands, aged and young animals showed equivalent discomfort during restraint (vocalizations and struggles- see Results, restraint stress). However, post-stress decline in water maze performance (Figure 1C), deep sleep loss/increased wake (Figure 2B), elevated CORT (Figure 5), and increased resting period body temperature (Figure 3C) all point to young subjects being more responsive than aged to that acute stress event. Young subjects also showed a hyperthermic response to water maze (also considered a stressor, Figure 3B) prior to restraint, while aged animals did not. Thus, our results point to decreased cognitive, sleep, thermal, and blood glucocorticoid secretion responses to acute restraint or water maze exposure in aged subjects. Molecular analysis revealed strong agreement with prior studies of age-related changes in the hippocampus, and a more complex interplay of this age-sensitive subset of genes with stress (please see Discussion below). These data may serve as a baseline for the construction of an understanding of the potentially more devastating influence of chronic psychosocial stress with age.

### Restraint model of psychosocial stress

Psychosocial stressors have been implicated in a variety of disorders in humans, from diabetes to depression (for review see Kajantie and Phillips, 2006). With age, the likelihood of experiencing a psychosocial stressor increases, and some common causes include the death of a loved one, loss of job through retirement, and social isolation due to care facility relocation. Psychosocial stress is operationally defined as stress induced by non-nociceptive stimuli. In rats, one accepted model is restraint, but others include abrupt and unpredictable light/sound/cage mate changes. We chose restraint as our stressor based on extensive prior literature (reviewed in Pawlyk et al., 2008) showing its effectiveness at activating the HPA axis in this animal model. Young and aged subjects showed similar struggle/vocalization responses to restraint although further studies of blood CORT levels during the restraint period may help to reveal whether restraint elicited a similar hormonal profiles in young and aged subjects during the stressor.

### Water maze

We trained animals on the visual cue task, and then surgically implanted them. After 2 weeks recovery and baseline EEG/EMG recording, animals were trained on the spatial water maze task. Aging deficits in water maze performance, as seen in multiple studies (Gallagher et al., 1993; Tombaugh et al., 2002; Blalock et al., 2003; Rowe et al., 2007; Bizon et al., 2009; Mawhinney et al., 2011; Vanguilder et al., 2012; Speisman et al., 2013) were clearly present here, particularly during early training sessions. Despite the 2-week intervening period between visual and spatial versions of the water maze, both young and aged animals appeared to remember platform location during the spatial task based on prior visual cue experience (Figure 1A- 3rd day vs. Figure 1B- 1st day). During the visual cue task, the spatial cues later destined to be used in the spatial task were already mounted around the water maze test area. Therefore, animals could have acquired spatial mapping data during the visual cue task. If so, then this may explain why young animals were performing at a high level even at the beginning of spatially cued training (Figure 1B). Future studies in which the spatial cues are removed during visual testing may help to evaluate the influence of cue pre-exposure to spatial task performance. If this interpretation is correct, then it would also follow that the young appear to be encoding spatial cues more effectively than aged. That said, despite the apparently stronger performance of young animals, it was the young spatial performance, and not aged, that appeared most disrupted by restraint (Figure 1C).

Aged and young training values were statistically indistinguishable by the third day of spatial training (Figures 1B1,B2), giving us a similar baseline for comparison of stress effects on the subsequent probe trial task. As a consequence, young animals, who reached that performance criterion more rapidly than aged, likely were “over trained” (i.e., they were at a performance plateau longer than the aged subjects). Thus, the negative influence of stress on water maze performance in young animals may be underestimated. In support of our working hypothesis that age acts as a stressor occluding the influence of subsequent stressors, young, but not aged animals, suffered a deficit in water maze performance post stress (Figure 1C). While this negative effect in young seems to point to an advantage of aging, we must consider that responding negatively to a stressor may be appropriate, and may be functionally necessary for longer-term hormesis and/or adaptive benefits that are potentially lost in non-responding older animals.

### Sleep architecture

Consistent with prior studies, aged animals showed less resting period deep sleep with age (Figure 2A). Further, young animals showed an aging-like shift in sleep architecture with stress exposure, increasing resting period wake, and losing REM and deep sleep. In human studies, increased wake/decreased sleep in response to stress are associated with poor coping strategies (Cespuglio et al., 1995; Morin et al., 2003). Further, both resting period REM and deep sleep are associated with improved memory and hippocampal function (Siegel, 2001; Kim et al., 2005; Ellenbogen et al., 2006; Vyazovskiy et al., 2008; Genzel et al., 2009; Born, 2010; Aleisa et al., 2011; Pace-Schott and Spencer, 2011; Zagaar et al., 2012). The loss of both REM and deep sleep with stress in young may be particularly disruptive to memory formation, while the increase in aged subjects' REM sleep (REM rebound) has been associated with a healthy stress response (Cespuglio et al., 1995; Gonzalez et al., 1995).

### Temperature and electrophysiological responses to stress

Body temperature measurements were taken to evaluate both diurnal zeitgeiber response, as well as to assess stress-induced hyperthermic response (Oka et al., 2001). Aged and young animals both show appropriate responses to light cues (Figure 3A), but the young, rather than the aged, showed a significant stress-hyperthermia after restraint (Figures 3C,D) that may be associated with their increased time awake. Surprisingly, a hyperthermic response was also present in young but not aged animals during water maze training prior to restraint stress (Figure 3B), highlighting aged subjects' blunted response to stressful events. The young animal hyperthermic response to water maze could be in response to temperature difference (water temperature = 26°C vs. rat body temperature = 36.8°C), suggesting that age-related acute stress blunting spans more stress modalities than just psychosocial.

Chronic stress' and stress hormones' influence on hippocampal structure and electrophysiology have been characterized (Joels et al., 1997; Conrad, 2010) although acute psychosocial stress' influence in aging hippocampus has been less well-studied. Here (Figure 4), aged animals are no different from their control counterparts in PP measures (Figures 4B,C), in agreement with prior work on stress hormone effects (Landfield et al., 1978a; Wu and Saggau, 1994). However, restraint significantly enhanced aged but not young input sensitivity (Figure 4A). Like increased REM sleep, it is intriguing to speculate that this may represent a beneficial response (Barnes et al., 1997; Thibault et al., 2001) that may provide compensation to aged subjects.

### Corticosterone measures

Aged subjects show a significantly smaller CORT response to stress than young (Figure 5). This seems to contradict the prevailing theory that CORT increases with age in both humans and rodents (Landfield et al., 1978a, 1986; Van Cauter et al., 1996; Porter and Landfield, 1998). However, these results are not unprecedented (for review see, Segar et al., 2009). Measures of the Fischer 344 rat diurnal rhythm report blood CORT highest early in the active period (200–300 ng/ml) and lowest early in the resting period (<100 ng/ml) (Dhabhar et al., 1993; Stohr et al., 2000). Our samples were taken approximately 4 h after peak CORT, putting our control results within acceptable ranges.

While the aging HPA axis is considered hyper-active (Meaney et al., 1992; Bizon et al., 2001; Herman et al., 2001; McEwen, 2008; Lupien et al., 2009), central components like excitatory amino acid level, corticotropin-releasing hormone, glucocorticoid receptor level, and CNS localized amplification of glucocorticoid action likely play a role (Lowy et al., 1995; Mabry et al., 1995; Herman et al., 2001; McEwen, 2001; Meyza et al., 2007; Yau and Seckl, 2012). As opposed to exaggerated glucocorticoid responses to non-psychosocial stressors (Sapolsky et al., 1986a) aged animals appear to have either the same or a blunted corticosterone or nerve growth factor response to psychosocial stressors like restraint (Stewart et al., 1988; Scaccianoce et al., 2000; Herman et al., 2001; Shoji and Mizoguchi, 2010; Garrido et al., 2012). Regarding diurnal variation in corticosterone, an elevated diurnal trough and either no change or a blunted diurnal peak in glucocorticoid secretion is also seen with aging (Sonntag et al., 1987; Goya et al., 1989; Carnes et al., 1994; Rowe et al., 1997; Gartside et al., 2003), suggesting a flattened diurnal rhythm.

### Gene expression

To get a closer look at molecular changes that might be at play, we investigated gene expression for 171 “aging panel” genes (Figure 6) with established age-related changes based on prior work (Blalock et al., 2003; Rowe et al., 2007; Kadish et al., 2009). The 101 up-regulated genes are associated with immune and inflammatory signaling while the 70 down-regulated genes have established roles in neuronal function. Here we discuss consequences of shifts in the expression of groups of genes rather than providing conjecture on the potential functional implication of individual gene expression changes. Thus, the molecular panel was used to determine (A) if the molecular profile of aging is identified (positive control), (B) whether this aging panel is sensitive to stress, and (C) if so, did stress modify young, aged, or both transcriptional profiles?

Clearly, we reliably detected the aging profile, with 93% of the genes showing directional agreement with prior studies. Many of those genes were significant with age regardless of stress status (Figure 7A inset). However stress itself appeared to have almost no influence on the transcriptional profile when evaluated from a conventional statistical perspective. That said, it is intriguing to note that a large cohort of genes were significant with age in the control, but not stress condition (42 genes).

This suggests that stress's effect on aging-sensitive genes is to constrict the influence of aging on these transcripts. If so, this could happen in several ways. For example, first, stress exposure could increase variability in gene expression, effectively reducing statistical discovery power among stressed subjects. Second, the transcriptional response could be CORT-driven (or driven by some CORT-associated change). Here a weaker CORT secretory response in aged (Figure 5), would lead to weaker CORT-driven gene expression in aged. Third, stress could selectively move the gene expression of young subjects toward aged values, or *vice versa*. And fourth, stress could regulate aging and young gene expression in an oppositional manner- driving young and aged gene expression values toward one another. The latter should be the least likely and would imply that stress/stress hormones have different effects in young and aged.

Because the following text focuses on direction of change and gene expression magnitudes that are, on an individual gene by gene basis, statistically non-significant, we feel it prudent to label this section as conjecture and restrict it to the Discussion. With that caveat, we found it surprising that the following appears to be the case. Among the 42 “control-only” genes, expression coefficients of variation were highly similar across groups (control 11.7% ± 1.1%; stressed 12.0% ± 0.9%). Further, the majority of these “control-only” aging genes (Figure 7B) do not show weaker responses in aged subjects. Instead, 79% show oppositional stress regulation with age. That is, if up-regulated by age, then stress both reduced expression in aged subjects and increased expression in young subjects (Figure 7B). Further, 91% of these “oppositional” genes were also identified as “oppositional” in a prior study identifying the age and glucocorticoid-sensitive transcriptional profile (Chen et al., 2013). Based on these observations, of the four possibilities laid out in the prior paragraph, our results support the fourth option, an apparent inversion of HPA-axis activity's action on a subset of aging-sensitive genes. This may parallel age-related shifts in the action of other steroid hormones such as estrogen (Sohrabji and Bake, 2006; Selvamani and Sohrabji, 2010; Rettberg et al., 2013; Singh et al., 2013).

### Summary and conclusions

In this study, rats showed characteristic aging changes seen across multiple mammalian aging systems, including worsened cognition (Gallagher et al., 1993; Salthouse, 1996; Joseph et al., 1999; Bickford et al., 2000; Erickson and Barnes, 2003; Tombaugh et al., 2005; Foster and Kumar, 2007; Gunn-Moore et al., 2007; Cotman and Head, 2008), disrupted deep sleep (Stone, 1989; Van Cauter et al., 2000; Cajochen et al., 2006; Mackiewicz et al., 2006; Espiritu, 2008; Tasali et al., 2008; Ancoli-Israel, 2009; Monjan, 2010), blunted synaptic sensitivity (Barnes et al., 2000; Thibault et al., 2001), and shifted hippocampal gene expression (Cotman and Berchtold, 2002; Blalock et al., 2003; Toescu et al., 2004; Galvin and Ginsberg, 2005; Rowe et al., 2007; Kadish et al., 2009; Burger, 2010). Similarly, young animals exhibited typical psychosocial stress responses, including disrupted sleep architecture (Marinesco et al., 1999; Van Reeth et al., 2000), worsened cognition (Stillman et al., 1998; Sandi et al., 2005; Nicholas et al., 2006; Kim et al., 2007; Park et al., 2008), stress-induced hyperthermia (Oka et al., 2001), and increased blood glucocorticoid levels (Sapolsky et al., 2000; McEwen, 2007). Further, Morris water maze and sleep architecture changes in young stressed animals recapitulate, at least in part, changes seen in normal aging. In conjunction with extensive prior work (Kerr et al., 1989, 1992; Bhatnagar et al., 1997; Oitzl et al., 2010) on exogenous glucocorticoid exposure in young animals, or long-term age-related consequences of pre- or perinatal stress exposure (Meaney et al., 1991; McEwen, 2002; Lupien et al., 2009; Eiland and McEwen, 2012), this work is consistent with one limb of the stress/allostatic load/glucocorticoid hypotheses of brain aging, that young subjects exposed to stress exhibit aspects of an aging-like phenotype.

However, if stress/glucocorticoid exposure leads to aging-like changes in young subjects' cognition and sleep, what happens to aged subjects exposed to the same acute stress? On the one hand, an acutely hyperreactive HPA axis might result in an exaggerated stress response (Kudielka et al., 2004). On the other, a chronically hyper-secreting HPA axis in aged, particularly elevated at the diurnal trough, may result in a blunted acute stress response. In fact, there is data to support this latter interpretation. Studies in humans and animal models have shown that chronic stressors such as asthma, depression and overcrowding all blunt HPA axis responses to acute psychosocial stressors (Buske-Kirschbaum et al., 2003; Gadek-Michalska and Bugajski, 2003; Tomiyama et al., 2011; Booij et al., 2013). Thus, aging/aging-related changes could be considered chronic stressors that blunt acute stress responses.

In this interpretation, age-related changes in central components like corticotropin releasing hormone (Segar et al., 2009) and localized glucocorticoid signaling (Chapman and Seckl, 2008), along with a flattened diurnal blood glucocorticoid oscillation (Sonntag et al., 1987; Goya et al., 1989; Carnes et al., 1994; Rowe et al., 1997; Gartside et al., 2003) may help to explain the reduced response of aged subjects to acute psychosocial stress. Alternatively, the blunted response could be due to adrenal fatigue (Van Den Eede et al., 2007; Nater et al., 2008; Kumari et al., 2009). In humans, adrenal fatigue is associated with chronic high stress environments like war (post-traumatic stress disorder) or constant pain (fibromyalgia, cancer), (Fries et al., 2005; Mease, 2005; Wu et al., 2012). However, here young subjects showed a hyper-thermic stress response prior to restraint while aged animals did not, CORT levels in controls were normal or even high, while low levels of cortisol are found in PTSD (Heim et al., 2000). Thus, hypo-responsiveness seems a more parsimonious explanation of the data.

Downstream consequences of this blunted acute response might include failed adaptation to repeated stress as seen in aged animals (Spencer and McEwen, 1997; McEwen, 1998). Further, reduced efficacy of hormesis-driven anti-aging manipulations (Masoro, 2000; Mattson, 2008) in late life, such as environmental enrichment, exercise and caloric restriction (Masoro, 2005; Pang and Hannan, 2013; Voss et al., 2013) and possibly dietary manipulation (Gemma et al., 2010), all may at least in part be explained by age-related stress hypo-responsiveness. Thus, aged animals may resist the cognition-worsening effects of acute psychosocial stress at the expense of their physiological capacity to adapt (McEwen, 2001, 2008). Despite over-activity of the HPA-axis having a bad reputation, and clear evidence of negative consequences, its hypo-response to acute psychosocial stress with age may also have long-term deleterious consequences.

## Conclusion

For the present work, then, aged control animals show characteristic changes in sleep architecture and cognition. Compared to aged, young animals show larger stress responses in water maze, sleep architecture, hyperthermia, and glucocorticoid secretion. Further, our assessment of REM sleep, transcriptional response and hippocampal input/output suggests that more than just being hypo-responsive to stress, on some measures aged subjects may show an opposite response, manifesting a more youthful profile with stress exposure. The most parsimonious conclusion is that aged animals are at least hypo-responsive to acute psychosocial stress. Because prior work demonstrates elevated HPA axis activity with age, we favor the interpretation that aging acts as a stressor whose presence occludes the influence of subsequent acute stressors. While this may be acutely beneficial for aged subjects, it may be deleterious in the long run, occluding stress adaptation and reducing the efficacy of “beneficial” stressors (eustress) such as caloric restriction, exercise, and environmental enrichment.

## Conflict of interest statement

The authors declare that the research was conducted in the absence of any commercial or financial relationships that could be construed as a potential conflict of interest.
